# Uncertainty Evaluation on a 10.52 GHz (5 dBm) Optoelectronic Oscillator Phase Noise Performance

**DOI:** 10.3390/mi12050474

**Published:** 2021-04-21

**Authors:** Patrice Salzenstein, Ekaterina Pavlyuchenko

**Affiliations:** Centre National de la Recherche Scientifique (CNRS), Franche-Comté Electronique Mécanique Thermique Optique Sciences et Technologies (FEMTO-ST) Institute, Université Bourgogne Franche-Comté (UBFC), F25000 Besançon, France; ekaterina2525@gmail.com

**Keywords:** optoelectronic oscillator, microwave signal, uncertainty, uncertainty analysis

## Abstract

This paper describes a prototype of an optoelectronic oscillator delivering a microwave signal with a power of 5 dBm at 10.52 GHz, promised to be compacted. It is evaluated in terms of its phase noise performance, and the associated ±2 dB uncertainty at 2 σ is calculated according to the international standards enacted for metrology.

## 1. Introduction

This paper aims to present an optoelectronic oscillator (OEO) intended to be compact. Currently, it exists on the table, but we have taken care to ensure that the various elements that constitute it can possibly be integrated in a compact volume, typically of the order of one liter. Let us go back in time a little and ask ourselves the question about when this type of oscillator was first introduced. OEOs were introduced 30 years ago [1]. OEOs based on optical resonators have already been developed [2], but their performances are still limited. We have thus chosen to develop an OEO based on an optical delay line. This OEO is characterized in this paper.

We can emphasize that OEOs are always the subject of work and improvement. Researchers have proposed different schemes to implement OEOs. Maleki explained the optoelectronic oscillator [3]. Hao et al. proposed a topology to break the limitation of mode building time in an OEO [4]. Ly et al. introduced coupled OEOs delivering a 90 GHz signal [5]. Integrated microwave optoelectronic oscillators were created by Tang et al. [6]. Chew et al. developed an OEO for a very precise temperature sensor [7]. Ge et al. proposed an open cavity OEO to generate random microwave signals up to 40 GHz [8]. Fan et al. created a photonic-delay line cross-correlation method for improving the used phase noise measurement of an OEO [9]. A tunable narrow linewidth photonic microwave signal based on an OEO was achieved by Lin et al. [10]. Chembo and his colleagues studied the nonlinear dynamics of miniature [11] and delay line OEOs [12]. Mode lock OEOs were studied by Wang et al. [13]. M. Jahanbozorgi et al. studied dispersion effects on the whispering gallery mode OEOs [14].

It is necessary to measure the performance of this oscillator and, above all, to assess the uncertainty associated with this level of performance. For the evaluation of the uncertainties associated with the measured phase noise levels, we based our work on a modern method of calculation, which is recommended by the International Bureau of Weights and Measures (BIPM). All of the measurements were subject to uncertainty, and a measurement result was complete only when the associated uncertainty accompanied a statement. The Joint Committee for Guides in Metrology (JCGM) is associated with BIPM. It is an organization from BIPM that prepared the “Guide to the Expression of Uncertainty in Measurement” (GUM). We used this guide for the evaluation of uncertainty, in order to maintain the correct standards.

This paper is divided into three main parts, which are themselves divided into subsections. We first present the materials and methods. Then, we give the results of the measurements obtained in terms of the phase noise performance. Finally, we proceed to the evaluation of the uncertainty associated with these results.

## 2. Materials and Methods

It is important to clearly define what we are measuring. First, we started by specifying what type of device under test (DUT) to characterize. Our goal was to be able to know the uncertainty on the validity of the results of the measurements on an oscillator, which is compact in the long term. For the sake of better understanding, it was easier to develop a DUT on the table in a laboratory. The goal was not so much to focus on the development of compactness at this stage, but rather to master the different elements of the DUT. When we had the DUT working well on the table, it was time to move on to the next step, which was the characterization of this DUT in terms of phase noise. To measure the noise performance of the DUT, we had two possibilities. We had a commercial bench and a bench made in the laboratory. Both of these measuring instruments had advantages and disadvantages. To begin with, on the one hand, the commercial bench was much easier to use for the operator in charge of the measurements. This commercial bench also made it possible to measure the noise quite far from the carrier, as will be seen later in this paper. On the other hand, the sensitivity of this bench was limited in terms of the measurable noise floor. Regarding the bench produced in the laboratory, it had the advantage of having a measurement noise floor significantly lower than the commercial bench. However, it did not allow measurements with an offset far enough from the carrier, and the use of this bench required more dexterity for the operator in charge of the measurements and more measurement time.

We divided this section in two subsections. The first consists of describing the DUT, while the second focuses on the phase noise measurement and explaining the type of instruments used for it.

### 2.1. Miniature OEO on a Table

In this section, we describe the DUT to be characterized. This DUT was an OEO undergoing miniaturization. For miniaturization for this type of OEO, we aimed to place it in conditions where, in the long term, the oscillator could fit in a volume of one liter. One liter is typically the volume that an on-board oscillator should have [15]. If this oscillator had a delay line, this goal of miniaturization would be too ambitious. However, the compactness of the element comprising the delay lines could be achieved by packaging a coil of fiber, as is done for gyroscopes [16]. For the other building blocks of the delay line-based OEO, compactness was ensured with patient 3D work on the OEO by interweaving the various components like the laser, modulator, and amplifiers, and providing compact power supply boards. The procedure followed here in the laboratory was not focused on compactness at this stage of the study. The compactness led to other constraints, such as the need to control the sensitivity of electromagnetism (EMC), which, in the case of an OEO, would be less impacted thanks to the optical aspect of a large part of the oscillation loop. Amplifiers and electrical components, on the other hand, are sensitive to EMC. It was not necessary to control the optical fiber through temperature, nor the possible stabilization of the laser by a feedback loop [17].

**Figure 1:**
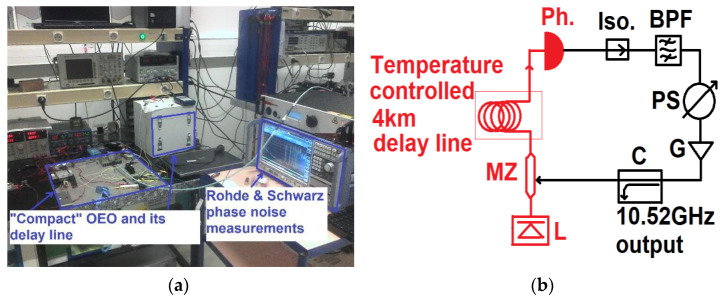
(**a**) Photo of the optoelectronic oscillator and the phase noise measurement commercial bench. (**b**) Optoelectronic oscillator (OEO): Optical and electrical elements are drawn in red and black colors, respectively. L—laser; MZ—Mach Zehnder modulator; Ph—photodetector; Iso—isolator; BPF—band pass filter; PS—phase shifter; G—microwave low noise amplifier; C—coupler.

For the OEO on the table, it delivered an output signal of 5 dBm at 10.52 GHz. It constituted a laser from RIO, model ORION driven by a 125 mA signal. Then, we had a modulator with an 11 GHz bandwidth, a 4 km optical fiber delay line, and a DSC40S Discovery photo-detector. In the electrical part of the loop, we had a 54 dB gain amplifier for the microwave signal, an X-band filter, an ARRA passive phase shifter, and a buffer amplifier (AML812-1901) at the lateral arm of a microwave coupler in order to extract the output microwave signal. The OEO is represented in Figure 1b.

It is appropriate to also say a few words about the gain of the amplifier in this type of oscillator. We recently celebrated a hundred years of Heinrich Barkhausen’s discoveries about the starting conditions of oscillators [18]. To adjust the gain of the microwave amplifier in the OEO oscillation loop, we proceeded with the help of a vector network analyzer (VNA).

This OEO is clearly visible in Figure 1. Here, we have a photograph of the OEO and the measurement bench, which served to characterize it. It was necessary to carry out this gain adjustment by working in an open loop. For a more general case, it was easy to understand that the losses in the delay lines and all of the optical devices also needed to be compensated in order to ensure the continuity of the oscillation phenomenon over time [19]. For the general case of the oscillator, other losses can occur. Simply, the propagation of an electrical signal in a circuit is not done without loss. We can therefore see that a certain number of losses must be compensated. Many other elements will generate losses of electrical or optical signal power in an oscillation loop. Filters, circulators, attenuators, isolators, power lines, phase shifters, and connectors also help to attenuate the signal strength in the oscillation loop. With knowledge of the reflectance at each point in the open loop oscillator circuit, we could verify the correct impedance match. This good adaptation is fundamental to avoid losses by reflection. When an oscillation circuit is correctly matched, i.e., adapted in impedance, if we are talking about an oscillator of the electric type, we can therefore look at the open loop with the transmission factor. One port of the VNA measured the received power level, and on another port, the analyzer delivered a transmitted power level. The difference in dB between the two power levels emitted and received corresponded precisely to what was missing, in terms of loss in the open loop of the oscillator under construction. The work then consisted of adding amplification elements in the loop. We inserted one or more amplifiers, taking the precaution to add insulators or filters if necessary. We needed to know the reflectance of each amplifier, as well as the noise factor, especially at the start of the amplification chain.

Figure 1a shows a picture of the OEO during measurements and Figure 1b describes the OEO.

### 2.2. Measurement Methods

**Figure 2:**
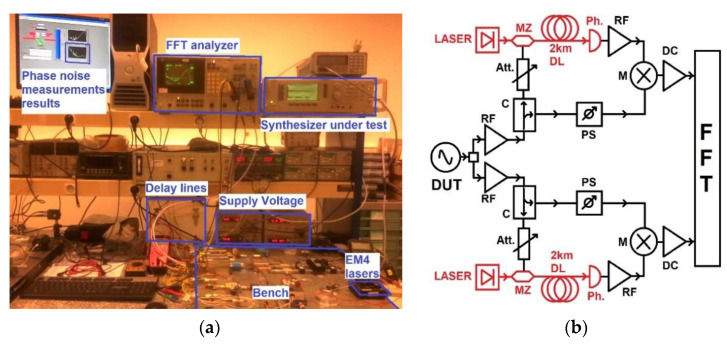
(**a**) Photo of the phase noise measurement bench developed at the laboratory while measuring the phase noise of a synthesizer under test. (**b**) Schematic view of phase noise measurement bench system using a double optical delay line. Optical elements and electrical elements are respectively drawn in red and black colors. DUT—device under test; MZ—Mach–Zenher modulator; DL—delay line; Ph—photodetector; M—mixer; DC—DC amplifier; RF—microwave amplifier; Att.—attenuator; C—directive coupler; PS—phase shifter; FFT—fast Fourier transform analyzer.

As mentioned previously, there are two ways to evaluate the performance of the DUT in terms of phase noise. Prior to the measurement, we calibrated the two systems with a commercial frequency synthesizer (Anritsu/Wiltron 69000B) with a declared phase noise of −105 dBc/Hz at 10 kHz of a 10 GHz carrier [20]. The results are shown in section “Result on the measurement of a known frequency synthesizer”. Figure 2 shows the setup of this measurement.

The bench is schematically represented in Figure 2b. This bench has two equal and fully independent channels. The phase noise of the DUT is determined by comparing the phase of the transmitted signal to a delayed replica through optical delay using a mixer. It converts the phase fluctuations into voltage fluctuations. Those fluctuations are sent to a double input FFT analyzer. The phase noise is determined thanks to an instrument based on two parallel arms pumped by two lasers at 1.55 µm. The signal delivered by the DUT is delayed by the optical fiber. It is divided in two branches. It allows the different noises from the devices in each bench to become uncorrelated. Then the noise floor can be considerably reduced by performing averaging of the incoherent sources. The noise floor is then reduced by β (expressed in dB) proportional to the m, and the number of averaging made is as follows:

β = 10 log(1/√(2m))(1)

Thus, the results were obtained using 2 km optical fibers delay lines and performing inter-correlation on 200 averaged, which gave a noise floor of −168 dBc/Hz at 10 kHz from the 10 GHz carrier. Next to this 10 GHz carrier, we measured −108 dBc/Hz at 100 Hz. These levels could be improved by increasing the number of averaging and the length of the delay line. Actually, with an FFT analyzer with a dual input like the HEWLETT-PACKARD model, HP3562A type, uncorrelated noise added by different components were averaged by inter-correlation following the previous relation (1). A 200 averaging took approximately 20 min. For a 500 averaging measure, it took 45 min and the obtained noise floor only decreased by 2 dBc/Hz. Cross correlation with 500 samples enabled a noise floor, typically in the order of £(f), of −170 dBc/Hz at 10 kHz from the X-band DUT carrier with a 2 km delay lines. We chose 2 km delay lines in the bench because it was a good compromise, as we were interested in characterizing the phase noise in the 10 Hz–100 kHz range. A 2 km delay line corresponded to a delay of τ = 10 µs, meaning a maximum Fourier frequency of 100 kHz.

Reference [21] describes the measurement system, but its uncertainty on phase noise was only estimated [22,23]; not all contributions were considered, for example the noise floor contribution. That is why it is necessary to describe the instrument in order to deduce the global uncertainty on the phase noise.

At this point in the paper, it is important to recall the general principles of the phase noise measurement using the measurement bench.

The short-term instability of the signal is characterized by a single sideband noise spectral density S_φ_(f) expressed in rad^2^/Hz. The IEEE [24] defines phase noise as £(f) = 10 Log [S_φ_(f)/2], expressed in units of decibels below the carrier per hertz (dBc/Hz). The phase noise determined with the instrument is defined as the ratio between the one-side-band noise power in one-Hertz bandwidth and the carrier power. If the mixer voltage gain coefficient is K_φ_ (volts/radian), then the mixer output rms voltage can be expressed as follows:

V^2^_out_(f) = K^2^_φ_│H_φ_(jf)│^2^S_φ_(f) (2)

where:

- K_φ_ is the mixer voltage gain coefficient expressed in volts/radian,
- │H_φ_(jf)│^2^ = 4·sin^2^(πfτ) is the transfer function of optical delay line,
- f is the Fourier offset frequency.

Equation (2) shows that the sensitivity of the bench depends directly on K^2^_φ_ and │H_φ_(jf)│. The first is related to the mixer and the second essentially depends on the delay τ.

We concretely used a fast Fourier transform (FFT) analyzer to measure the spectral density of noise amplitude V^2^_out_(f)/BW, where BW is the bandwidth used to calculate V_out_(f)/BW. The phase noise of the DUT is finally defined by Equation (3) and by taking into account the gain of DC amplifier G_DC_, as follows:

£(f) = 10 Log ([V^2^_out_(f)]/[2K^2^_φ_.│H_φ_(jf)│^2^ G^2^_DC_ BW]) (3)

## 3. Results

This part is divided into two subsections. The first will briefly describe the results made with the measurements on a known frequency source; in this case, a frequency synthesizer, which we talked about in the previous section. The second subsection relates to the measurement of the DUT chosen as a pre-prototype of a compact OEO, this time again developed on a laboratory table, but which has the desired characteristics in terms of power or frequency delivered.

It is important, in our opinion, to give some details on the way in which the measurement results were obtained. For the frequency synthesizer serving as a comparison, for the OEO set up, and also the commercial measurement bench and the measurement bench specific to the laboratory, it is necessary for them to all have similar conditions of use. First of all, we operated with all instruments switched on for one hour minimum before use to ensure their voltage and temperature stability. Then, 50 Ohm load terminations were fixed on all unused outputs or inputs. The DUT should also be stabilized in temperature. The cables were not changed so as to avoid undesired effects. The Mach Zehnder modulator (MZM) was set at its working point. This means that the researched optical out power of the MZM needed to be in a linear zone regarding the V_π_ bias voltage. The optical power of each laser diode was adjusted to K_φ_, P_RF_, and V_photodetector_. The bench was calibrated with a known source such as a synthesizer. Then, the DUT was substituted to the reference source during the measurement.

In general, it is prudent when we wish to carry out a comparison between two measuring means to use comparable conditions. Here, we used the same room. The temperature was the same and the devices were all turned on for at least an hour before taking the measurement.

### 3.1. Result on the Measurement of a Known Frequency Synthesizer

Table 1 gives the results of these measurements. From these first measurement results, we concluded that our system could give the same phase noise results for a commercial synthesizer. We underlined that this synthesizer was noisier than the noise floor of the commercial instrument (at −125 dBc/Hz). Despite our bandwidth being limited to 100 kHz (τ = 10 μs) for our laboratory bench, the measured phase noise was the same with the Rohde and Schwarz bench, and the results were consistent.

### 3.2. OEO Phase Noise Characterization Results Chacterization Methods

The result of the phase noise spectral density measurements at working Fourier frequencies between 2 × 10^3^ and 4 × 10^4^ Hz are given in Table 2. The results of the phase noise measurements were a little different depending on the bench used for the phase noise characterization of the OEO. The measurement results differed between the same OEO measured by the commercial Rohde and Schwarz instrument and our system, because of the noise limitation at −125 dBc/Hz for the R&S instrument. The commercial bench was a Rohde and Schwarz model, type FSW, and did not have the option for low phase noise. The OEO presented a minimum of −145 dBc/Hz at 3 × 10^4^ Hz from the X-band carrier. This was mainly because the level of performance of the OEO was better than the bench possibilities. Fortunately, the OEO was no better than the possibilities of the bench developed in the laboratory.

We proceeded according to reference [25] to estimate the flicker frequency modulation (FFM) floor of our OEO. We made the hypothesis that the OEO delivered a sinusoidal signal. In Table 2, if we consider that the slope of the curve is approximatively in f^−3^ at 10 kHz from the 10.52 GHz carrier, we can then calculate the Allan deviation σ_y_(τ) in the time domain [26]—which corresponded to an FFM floor expressed in relation (4) to the folllowing:

σ_y_(τ) = √(2Ln2 h_−1_) (4)

where h_−1_ is the power coefficient in the Allan variance power-law response. It means the OEO had an FFM floor of 5 × 10^−11^ in terms of its Allan deviation. It corresponds to the minimum stability of its frequency in the time domain.

We investigated the limit of our measurement bench developed in the laboratory. The background phase noise of the bench was determined after performing 500 averaged with the cross-correlation method, when removing the 2 km optical delay line of the bench [22]. In this case, the phase noise of the 10 GHz synthesizer is rejected. The noise floor (without optical transfer function) was better by −150 and −170 dBc/Hz at 10^1^ and 10^4^ Hz, respectively, compared with the 10 GHz carrier. When the optical fiber was introduced, the noise floor of such a system was up to −90 and −170 dBc/Hz at 10^1^ and 10^4^ Hz from the 10 GHz carrier. The determined noise floor of the bench ensured that the phase noise measured with this bench for a DUT was trustful. These results showing the evaluation of the measurement floor are given in Table 3. Analogously to what we wrote about Table 2, it means the bench had an FFM floor of 5.3 × 10^−13^ in terms its Allan deviation. So, this bench was able to measure microwave oscillators with a frequency stability no better than 5.3 × 10^−13^.

## 4. Discussion about the Uncertainty

Investigating the uncertainty calculation is an old challenge of scientists working on phase noise. It firstly concerns the knowledge of the experimentally determined phase noise close to the carrier with a negative slope of Sφ(f) versus the Fourier frequency noted f, and secondly, it concerns the determination of the ground noise f0 far from the carrier, mostly dependent from the power inside the loop with an approximation of kT/P, where k is the Boltzmann constant, T is the temperature, and P is the power. Fred Walls and his colleagues from NIST described the principle of phase noise and its calculation [27,28]. In 2010, Won-Kyu Lee from Korea explained their work concerning uncertainty calculation [29]. In 2013, sources of uncertainties in an uncertainty evaluation were discussed by Shinya Yanagimachi and his colleagues from Japan [30]. Several phase noise measurement techniques were investigated by Ulrich L. Rohde and Ajay K. Poddar from Germany in 2013 [31]. For the uncertainty calculation, we proceeded similarly to the determination of the uncertainty for a purely microwave setup [32].

The uncertainty was calculated according to the main guideline delivered by the Bureau International des Poids et Mesures (BIPM) in the guide “Evaluation of Measurement Data—Guide to the Expression of Uncertainty in Measurement” [33]. Actually, we followed a modern approach to express uncertainty in measurement [34]. The uncertainty in the results of a measurement consist of several components, which may be listed as two categories according to the way in which their numerical value is estimated.

It is interesting to consider how the elementary terms are grouped together for the calculation of the final uncertainty. We can see that we are dealing with two main categories of elementary uncertainty terms.

The first category of terms of uncertainty is called “type A”. These terms are evaluated by statistical methods such as reproducibility, repeatability, special consideration about Fast Fourier Transform analysis, and the experimental standard deviation. The components in category A are characterized by the estimated variances.

The second family of uncertainty contributions are evaluated by other means. They are called “type B”, and because various components and temperature control, experience with or general knowledge of the behaviour and properties of relevant materials and instruments, manufacturer’s specifications, data provided in calibration and other certificates (noted BR), their uncertainties assigned to reference data taken from handbooks. The components in category B should be characterized by quantities, which may be considered as approximations to the corresponding variances, the existence of which is assumed.

We are getting to the significant part about uncertainties. We must now examine each of the elementary terms.

### 4.1. Statistical Contributions

Repeatability (A1): It is the variation in measurements obtained by one person on the same item and under the same conditions. Repeatability conditions include the same measurement procedure, the same observer, and the same measuring instrument used under the same conditions, repetition over a short period of time, and at the same location. We automatically performed 4 to 10 measurements with the fast Fourier transform (FFT) analyzer. The elementary term of uncertainty for repeatability e_Rep_ was experimentally found to be equal to 0.3 dB for 4 measurements and 0.2 dB for 10 measurements at 1σ. Its probability distribution was normal (Gaussian). A1 was thus deduced with a 0.682 at 1 σ (where σ is the standard deviation).

Reproducibility (A2): Measurements are performed by the same operator. There are no changes caused by differences in the operator behavior. All components and devices are dedicated to the instrument and none of them are replaced. This term was selected as zero.

Finally, statistical contribution can be considered as follows:

A = √(ΣAi)(5)

According to Equation (5), it can then be considered that the whole statistical contribution is better than 0.69 dB.

### 4.2. Contributions Evaluated by Other Mean

BR: The phase noise measurements are not referenced to a standard, as the method is intrinsic. So, the data provided in calibration and other certificates, noted as BR, are not applicable. Thus, we took 0 dB as a good approximation of BR.

Temperature variation (BL1): Temperature variation in the laboratory is in the range 21–25 °C. The maximum variation is ±2 °C. Its influence on the phase noise of the DUT is e_Temp_ = 10Log(298/296) = 0.0292 dB. This distribution is rectangular. It is important to clarify that these variations are slow variations. We deduced that BL1 = 0.292/√3 = 0.017 dB.

Phase conversion factor (BL2): this factor K_φ_ is determined by the generation of a low frequency beat note signal to be sent to the FFT analyzer. It corresponds to the slope at the zero crossing point according to the gain G of the low noise amplifier.

K_φ_ = ΔV/(ΔΦ.G) (6)

where

ΔΦ = (t_2_ − t_1_).2π/T (7)

T is the period of the beat note frequency signal, t_2_ and t_1_ are the times when the beat note frequency signal is set at zero and at ΔV, respectively, in the order of 1 ms. t_2_ is in the range of 32 µs and t_1_ << t_2_. By introducing Equation (7) into (6) and considering that ΔΦ is very small, we deduce the following:

20LogK_φ_ = 20Log ΔV + 20LogT − 20LogG − 20Logt2 − 20Log(2π) (8)

We can then calculate e_ΔV_, e_T_, e_G_, and e t_2_, i.e., each contribution of ΔV, T, and t_2_—to the uncertainty on K_φ_: e_ΔV_ = 20Log[(ΔV + e_specΔV_)/ΔV], where e_specΔV_ = 0.1% is the specification on the determination of ΔV. So, e_ΔV_ = 0.01 dB. e_T_ = 20Log[(t_2_ + T_max_)/t_2_], where T_max_ = 0.1 µs is the sampling interval of the FFT. So, e_T_ = 0.027 dB. e_G_ = 20Log[(G + e_det-of-G_)/G], where e_det-of-G_ = 1% is the maximum relative error on the determination of the gain. So, e_G_ = 0.086 dB.

From these considerations the elementary uncertainty term on K_φ_ = is deduced as e K_φ_ = √(e_ΔV_^2^ + e_T_^2^ + e_G_^2^). So, e_Kφ_ = 0.086 dB. By applying a rectangular distribution, we deduce that BL2 = e _K__φ_/√3 = 0.053 dB.

Noise floor contribution (BL3): As shown in Table 3, the noise floor of the instrument is −90 and −170 dBc/Hz at 10^1^ and 10^4^ Hz, respectively, from the X-band carrier. For a measured noise of −80 dBc/Hz and −140 dBc/Hz at 10^1^ and 10^4^ Hz, the maximum error is e_NoiseFloor_(10^4^) = 0.001 (corresponding to −30 dB) and e_NoiseFloor_(10^1^) = 0.1 (corresponding to −10 dB), respectively, and for a DUT at −80 dBc/Hz and −140 dBc/Hz at 10^1^ and 10^4^ Hz respectively. We deduce BL3 by taking a rectangular distribution of BL3(10^4^) = 0.001/√3 =0.00058 dB and BL3(10^1^) = 0.058 dB. This last value is reasonable to be used in the calculation.

Resolution of instruments (BL4): it is determined with a rectangular distribution by the value read on each voltmeter when we need to search the minimum and maximum for the modulator, but also for a power meter. The resolution is then no worse than 0.1 dB. BL4 = 0.1/√3 = 0.058 dB.

Contribution of the use of automatic/manual range (BL5): we can deduce from the experimental curves that this influence is no more than 0.02 dB. BL5 = 0.02/√3 = 0.012 dB.

Contribution of the lasers to the noise (BL6): The relative intensity noise (RIN) of lasers is related to the ratio between the average of the square of the fluctuation optical power (δφ) on the square of the average optical power φ_0_^2^.

RIN(ω) = <│δφ │^2^ >/φ_0_^2^ (9)

where ω is the pulsation. RIN generally presents a floor until the Fourier frequency is equal to the relaxation frequency of the laser. Then, the noise decreases. This relaxation frequency is generally in the range of a few Mega Hertz. Datasheet of the EM4 1550 nm distributed feedback (DFB) laser indicates an RIN no worse than −150 dB/Hz at 10 GHz. The contribution of these DFB lasers is eliminated by cross correlation, so we can consider that BL6 = 0.

Total contribution of BL = ΣBLi is the arithmetic sum of each elementary contribution. It was determined to be BL = 0.017 + 0.053 + 0.058 + 0.058 + 0.012 + 0 dB =0.198 dB.

### 4.3. Estimation of the Global Uncertainty of This System

Uncertainty at a 1 σ interval of confidence is calculated as follows:

u_c_ = √(A^2^ + BR^2^ + BL^2^) (10)

We deduce from Equation (10) that the uncertainty at 1 sigma, noted as u_c_, is better than √(0.69^2^ + 0.20^2^) dB. Its leads to a global uncertainty of ±0.72 dB at 1 σ. For convenience and to keep an operational uncertainty in case of the degradation or drift of any elementary terms of uncertainty, it is wise to degrade the global uncertainty. This is why we choose to keep U = ±2 dB at 2 σ for a common use of the phase noise optoelectronic instrument, instead of ±1.44 dB at 2 σ. This final uncertainty is defined at 2 σ, according to the empirical rule 68.27% at 1 σ is not enough, but 95.45% at 2 σ is more efficient for a normal distribution in statistics.

## 5. Conclusions

To conclude this paper, we can indicate that we have characterized the OEO in terms of phase noise. The signal delivered at 10.52 GHz with an output power of 5 dBm presents a relatively good performance in terms of phase noise, with a minimum of −145 dBc/Hz at 3 × 10^4^ Hz from the carrier. The associated uncertainty is better than ±2 dB at 2 σ. This result is encouraging for an OEO produced on a table, and which, can potentially be rearranged into a compact prototype that fits in a volume of one liter.

## Figures and Tables

**Table 1:** Phase noise of an Anritsu synthesizer with an output power of 10 dBm at 10 GHz, measured by our instrument and by the commercial Rohde and Schwarz (R&S) bench.

Offset to the 10 GHz Carrier Fourier Frequency (Hz)	Measure with R&S Bench Phase Noise (dBc/Hz)	Measure with Our Bench Phase Noise (dBc/Hz)
10^1^	−60	−60
10^2^	−88	−88.5
10^3^	−97	−97
10^4^	−93	−93
10^5^	−108	−108
10^6^	−137	NA ^1^

^1^ Non applicable. This value is not measured by our bench, because 1 MHz is not in its bandwidth.

**Table 2:** Phase noise of an OEO with an output power of 5 dBm at 10.52 GHz, measured by our instrument and by the commercial Rohde and Schwarz (R&S) bench.

Offset to the 10.52 GHz Carrier Fourier Frequency (Hz)	Measure with R&S Bench Phase Noise (dBc/Hz)	Measure with Our Bench Phase Noise (dBc/Hz)
2 × 10^3^	−100	−100
4 × 10^3^	−109	−112
6 × 10^3^	−115	−118
10^4^	−119	−130
2 × 10^4^	−125	−140
3 × 10^4^	−125	−145
4 × 10^4^	−123	−141

**Table 3:** Noise floor of the instrument.

Offset to the 10 GHz Carrier Fourier Frequency (Hz)	Noise Floor Determined with 500 Averages with an Anritsu Synthesizer at the Input of Our Bench £(f) in dBc/Hz
10^1^	−90
10^2^	−119
10^3^	−145
10^4^	−170
3.5 × 10^5^	−170
9.5 × 10^5^	−160

## Data Availability

Data are not publicly available due to restriction access.
